# Introductory Lecture to the Course on Pathological Anatomy at the University of Philadelphia

**Published:** 1871-11

**Authors:** 


*Introductory Lecture to the Course on Pathological Anatomy at the University of Philadelphia.* By Joseph G. Richardson, M.D.

In the course of this lecture, Dr. Richardson asserts that a great many pathological changes in the human body are due to hereditary tendencies, and inheritances of microscopical peculiarities. He thinks that, "just as the evolutionist philosopher may claim to have obtained a clue to the mysterious development of organic life, through past ages, upward towards a higher type, in that pregnant phase, 'the survival of the fittest,' so we, as physicians, may discover a universal key to those problems of hereditary disease among which our life-work is cast, in its anthithesis, the funeral fiat which decrees for individuals, families, and races, alike, *the extinction of the unfit.*"

